# Evaluation Research of the Effects of Longitudinal Speed Reduction Markings on Driving Behavior: A Driving Simulator Study

**DOI:** 10.3390/ijerph13111170

**Published:** 2016-11-23

**Authors:** Han Ding, Xiaohua Zhao, Jianming Ma, Jian Rong

**Affiliations:** 1 College of Metropolitan Transportation, Beijing University of Technology, Beijing 100124, China; dinghan86@emails.bjut.edu.cn (H.D.); jrong@bjut.edu.cn (J.R.); 2 Beijing Key Laboratory of Traffic Engineering, Beijing University of Technology, Beijing 100124, China; 3 Traffic Operations Division, Texas Department of Transportation, Austin, TX 78701-2483, USA; jianming.ma@txdot.gov

**Keywords:** traffic safety, longitudinal speed reduction markings, driving behavior, interchange connector, driving simulation

## Abstract

The objective of this paper is to explore the effects of longitudinal speed reduction markings (LSRMs) on vehicle maneuvering and drivers’ operation performance on interchange connectors with different radii. Empirical data were collected in a driving simulator. Indicators—relative speed change, standard deviation of acceleration, and gas/brake pedal power—were proposed to characterize driving behavior. Statistical results revealed that LSRMs could reduce vehicles’ travel speed and limit drivers’ willingness to increase speed in the entire connector. To probe the impacts of LSRMs, the connecter was split into four even sections. Effects of LSRMs on driving behavior were stronger in the second and the final sections of connectors. LSRMs also enhanced drivers’ adaptability in the first three quarters of a connector when the radius was 50 m. Drivers’ gas pedal operation would be impacted by LSRMs in the entire connector when the radius was 50 m. LSRMs could only make drivers press brake pedal more frequently in the second section with 80 m and 100 m radius. In the second quarter section of a connector—from the FQP (the first quartile point) to the MC (the middle point of curve)—LSRMs have better effects on influencing vehicle maneuvering and drivers’ operation performance.

## 1. Background

**Figure 1:**
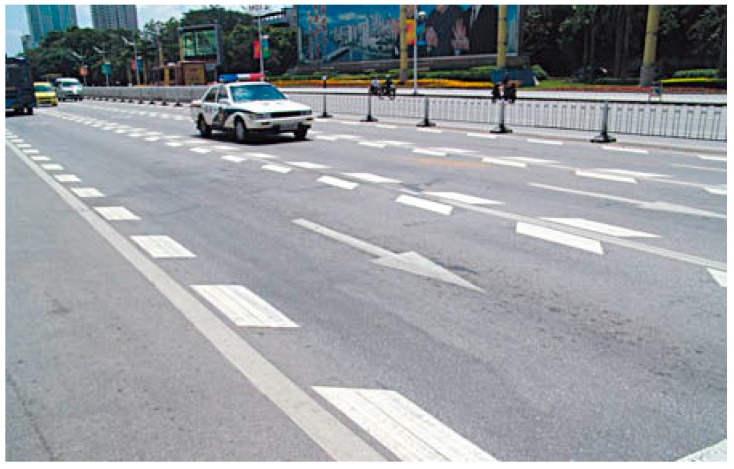
Longitudinal speed reduction markings (LSRMs) in China.

According to [1], speeding accounted for 11.7 percent of all traffic fatalities in China in 2012. To address this issue, speed control devices are installed at sites where speeding-related traffic crashes are more likely to happen. Among all kinds of speed control devices, longitudinal speed reduction markings (LSRMs) are widely used on urban expressways and highways [2]. In Beijing, LSRMs are installed on interchange connectors by the Beijing Traffic Management Bureau in engineering practice (see Figure 1). Meanwhile, the 2009 U.S. *Manual on Uniform Traffic Control Devices* (the 2009 MUTCD) also offers a series of detailed requirements for the use of speed reduction markings (SRMs). Since the design and application of SRMs are unique in China, it is necessary to evaluate the effectiveness of these SRMs specified in the *China National Standard of Traffic Control Devices*.

It is still unclear whether LSRMs could reduce or influence vehicle speeds and to what extent SRMs could reduce vehicle speeds on interchange connectors. Therefore, numerous research studies have been conducted to evaluate the effectiveness of SRMs or other traffic control devices in interchange connectors or curves. For example, Maroney and Dewar [3] investigated the effects of transverse lines, which were regarded as a means of reducing speeding behavior. Charlton [4] evaluated the perceptual and attentional effects of three types of curve warnings on driver’s speed selection. Similarly, some studies evaluated the effectiveness of SRMs in terms of speed, or other indicators related to speed. Gates et al. [5] researched the effectiveness of transverse bar pavement markings on freeway curves. Katz [6] evaluated the effects of SRMs through field tests and driving simulator experiments. Ding et al. [7,8,9] studied the effectiveness and adaptability of LSRMs and transverse speed reduction markings (TSRMs) on downhill sections, from the perspective of vehicle maneuvering and drivers’ operation performance. In other words, the effectiveness of SRMs was validated by comparing the speed data recorded before and after the SRM segment in previous studies. However, although many research studies have focused on the effectiveness of SRMs, the research related to this specific road condition—the interchange connector on an urban expressway—is rare, and no guidance can be referred to support the installation of LSRMs under this condition.

In addition, LSRMs are nonintrusive speed control devices, which are not enforceable; this means that sometimes LSRMs might fail to prompt a change in vehicle speeds. In fact, according to the national standard [2], the main purpose of LSRMs is to alert drivers to reduce speeds. If a driver perceives the existence of LSRMs and decides to slow down, we can say that SRMs are effective. From this perspective, some researchers turned to study the impacts on driving behavior. For instance, Kaber et al. [10] pointed out that a driver’s conscious control is associated with the driver’s reactions to roadway conditions. Zheng [11] stated that LSRMs could cause some visual illusions for drivers, suggesting that the lane becomes narrower. Those influences on drivers’ perceptions cause them to decelerate. Comte and Jamson [12] studied the impacts of transverse bars on driver performance, workload and acceptability.

Furthermore, driving behavior is highly associated with perception and judgment. If SRMs make drivers decide to slow down, drivers will execute related operating performances. Among all kinds of operating performances, letting off the gas pedal and/or pressing the brake pedal can directly reduce vehicle speed. Consequently, researchers can validate the effectiveness of SRMs on drivers’ judgment by measuring drivers’ gas and brake pedal performance. For instance, Rakauskas et al. [13] used gas pedal position to measure the effects of cell phone conversations on driving performance. Mulder et al. [14] illustrated drivers’ neuromuscular measurements of gas pedal positions and how much force drivers applied to the pedal when driving in the simulator. Ding et al. [7,9] chose deceleration operation frequency and operating power as indicators to evaluate the effectiveness of both kinds of SRMs, concluding that TSRMs are more effective than LSRMs in influencing drivers’ operation performance on downhill sections, such as making drivers press harder on the brake pedal. In [15], researchers used gas pedal use to measure how driving simulator configurations affected driver’s behavior and control performance. Saffarian et al. [16] introduced some indicators to measure braking behavior, such as the maximum brake pedal displacement and maximum brake pedal displacement.

Existing research to evaluate the effectiveness of SRMs has usually been performed through field studies to acquire data reflecting vehicle operation status (including speed, acceleration, etc.). Field tests can ensure the reliability of speed and operating performance data; however, it is difficult to evaluate changes in speed or performance due to the presence of SRMs because vehicle operation and drivers’ performance are actually affected by numerous factors, such as the presence of SRMs, roadway alignment, and traffic volume and density. Besides, since the objective of this paper is to explore the effects of LSRMs on driving behavior, a simulation study not only collects speed data, but also records drivers’ operating performance data, such as gas and brake pedal use, steering, and shifting gears more directly and safely, compared to field tests. Moreover, the driving simulator study could help us better control various variables, isolate other confounding factors and allow us to observe the effect of SRMs alone on vehicle status or drivers’ performance, and there are a series of current studies focusing on driving simulator method [17,18,19].

Since the validity of driving simulator will influence the results and conclusions of this research, a series of validation studies were performed. Xu [20] validated the effectiveness of the Beijing University of Technology driving simulator, and corroborated that the driving simulator is effective in terms of capturing drivers’ physiological and psychological parameters. Ding et al. [7,8,9] also validated the effectiveness of the driving simulator on travel speed in downhill segments equipped with SRMs, compared to speed data collected in the field. According to [7,8,9], the driving simulator had relative effectiveness in terms of simulating travel speeds, and the speed variation trend in the downhill segments equipped with SRMs was highly similar to the trend in a real road environment. Although all test routes in field tests that were described in those references consisted of tangents and curves, the validation of driving simulator corresponding to interchange connectors will be put forward.

Referring to previous studies, the objective of this paper was to evaluate the effectiveness of LSRMs on vehicle maneuvering and drivers’ operation performance on interchange connectors with different radii, through a driving simulator experiment. Since there were insufficient studies or regulations about the effects of LSRMs on interchange connectors in China, in terms of vehicle maneuvering and drivers’ operation performance, which led to various kinds of misuse in engineering practice, this research was the first study in China. If such works would be stretched to engineering practice, we will recruit drivers to drive in interchange connectors under real road surroundings with instrumented vehicle, use real-world data to judge the deployment of LSRMs, and validate driving simulator at the same time.

## 2. Methods

### 2.1. Subjects

This experiment was conducted in March 2013, and female subjects accounted for a small number of the total participants in this experiment, based on the demographic characteristics of licensed drivers in China at that time [21]. Meanwhile, the goal of this research was to lay a foundation for developing guidelines for SRMs installation, and younger drivers tend to drive more aggressively and would benefit more from speed reduction countermeasures. Therefore, younger participants were mainly considered for this research.

While it would have been desirable to have a higher number of subjects, it is common for simulator studies to obtain a small sample size, due to the high resource demands. For example, research into the effects of shoulder width, guardrail and roadway geometry on driving behavior was performed with 22 participants [22]; research into the influence of roadside infrastructure on vehicle operation was also conducted with 22 participants [23]; a study aiming at the effects of additional road markings on speed limits was performed with 30 participants (27 males and 3 females) [24]; and a study focusing on the safety effects of enhanced road markings was performed with 25 participants [25].

In recruitment, drivers were required to drive in a scenario with complex road elements in driving simulator, so as to decide who will not feel sick in driving simulator, and such drivers were recruited as participants in this experiment. Twenty-seven male and three female subjects, ages 18 to 42, with an average age of 24.8 years and an average driving experience of 3.3 years, were recruited by advertisement. Specifically speaking, percentages of participants in different age groups (ages 18–25, 25–30 and over 30) were 45%, 45% and 10%, respectively. To capture subjects’ actual reactions to the presence of LSRMs, participants were not informed of the purpose of the study. All subjects gave their informed consent for inclusion before they participated in the study.

### 2.2. Apparatus

Technical parameters of a fixed-base driving simulator used in this experiment have been introduced [7,8,9]; the real-time data were collected, including operating performance data (e.g., accelerating, decelerating and steering). The values of gas and brake pedal use range from 0 to 1, representing the press intensity of these two pedals. For instance, “0” denotes that the pedal is not pressed, while “1” represents that the pedal is pressed to its full depth. The data acquisition frequency is 30 Hz, and the virtual scenario was projected onto three large screens, providing a 130° field of view. Moreover, the driving simulator is able to generate various sensory effects for participants, such as visual, auditory and tactile effects.

### 2.3. Scenarios

**Figure 2:**
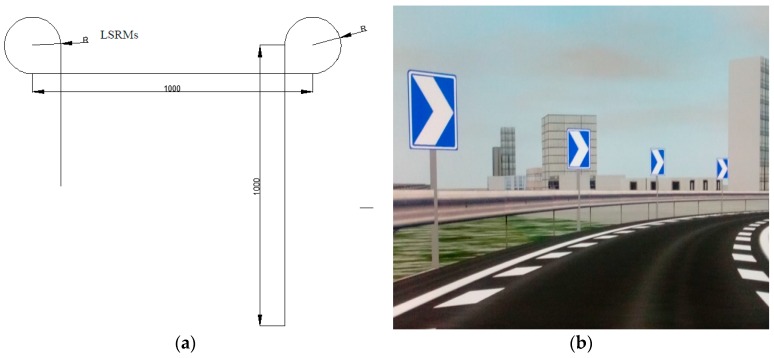

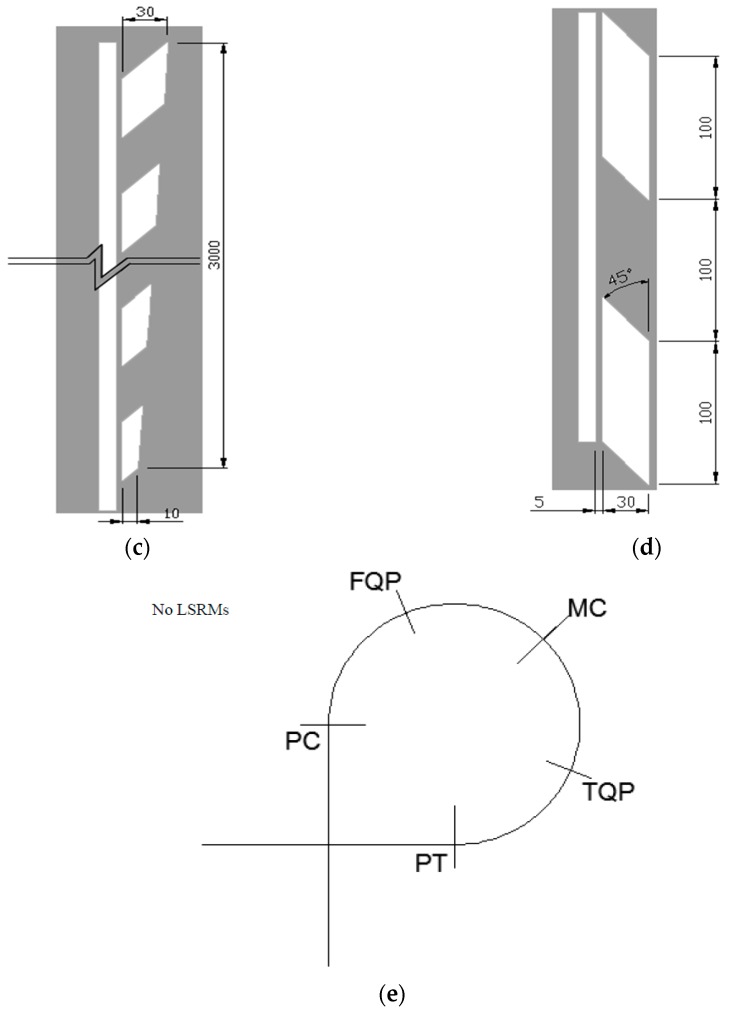
Scenario design: (**a**) Profile graph of scenarios; (**b**) four segments in one connector; (**c**) transition taper of LSRMs (in centimeters); (**d**) detailed designs of LSRMs (in centimeters); and (**e**) LSRMs designed in the experimental scenario.

As mentioned in the national standard, LSRMs are actually placed on urban expressway interchange connectors. According to the Chinese industrial standard *Code for Design of Urban Road Engineering* (CJJ37-2012) [26], the radii of interchange connectors range from 50 m to 100 m. Therefore, this study designed a total of six experimental segments {i.e., 2 (No LSRMs vs. LSRMs) × 3 (radii of 50 m vs. 80 m vs. 100 m)}, and three virtual scenarios were created. Each scenario featured an eight-lane, divided urban expressway, with two one-kilometer tangents regarded as transition sections. Furthermore, each scenario contained two connectors of interest, one without LSRMs and one with LSRMs, respectively (see Figure 2a,b). Additionally, the radii of the interchange connectors in one scenario were the same. Scenarios 1 to 3 were labeled the 50-R scenario, 80-R scenario, and 100-R scenario, representing different radii of interchange connectors. Detailed designs of LSRMs are shown in Figure 2c,d, and alignment parameters of virtual scenarios are listed in Table 1.

To thoroughly observe the effects of LSRMs on driving behavior in the interchange connectors, we divided the interchange connector into four even segments, shown in Figure 2e. PC means the “Point of Connector”, which is the beginning point of the connector; FQP refers to the “First Quartile Point”; MC represents the “Middle of Connector”; TQP is the “Third Quartile Point”; and PT means the “Point of Tangent”, which is the ending point of the connector.

### 2.4. Procedures

Subjects were required to fill out a questionnaire prior to the test, which recorded their basic information (such as age, gender, driving experiences, etc.) as well as their physiological and psychological conditions. Then subjects were required to perform a practice drive for 5–10 min on a specific alignment in order to become familiar with the driving simulator.

After the practice drive, subjects participated in the formal driving experiment, in which the vehicle maneuvering and drivers’ operation performance data were collected. Orders of three scenarios for each subject were randomly assigned. Each scenario was driven once, and subjects were given a brief rest between different scenarios. The entire driving experiment lasted approximately 20 to 30 min. When the formal experiment was finished, each subject was requested to fill out a questionnaire to report his or her subjective evaluation of the driving simulator, and his or her physiological and psychological status after the experiment. According to information revealed from this questionnaire, two subjects felt sick after the formal test, occupying 6.7% of all subjects.

## 3. Analysis and Results

In this paper, effects of LSRMs on driving behavior will be discussed. Four indicators were selected: relative speed change, standard deviation of acceleration, gas pedal power and brake pedal power.

*Relative speed change* was used to evaluate the decelerating ability of LSRMs, which was defined as:

(1) $$\theta =\frac{v_{2}-v_{1}}{v_{1}}\;.$$

where $v_{1}$ is the entering speed, denoting the vehicle speed upon the entering point of each section; $v_{2}$ is the exiting speed, denoting the vehicle speed upon the leaving point of each section; and θ is the relative speed change.

If this indicator was negative, it meant that the vehicle decelerated while traveling in the section, and vice versa.

*The standard deviation of acceleration* was used to represent the process of speed variation, the steadiness of driving status and the adaptability of LSRMs through the downhill section. A high standard deviation indicated that drivers adjusted traveling status heavily while driving through the downhill sections with LSRMs; that is, drivers might feel nervous or uncomfortable, suggesting the worse adaptability of LSRMs.

*Gas pedal power* and *brake pedal power* were used to evaluate the effects of LSRMs on driver’s operation performance. Both indicators not only measure the intensity of drivers’ pressure on the gas pedal and brake pedal, but also consider the duration and frequency of drivers’ use of the gas and brake pedals [9].

### 3.1. The Entire Connector

**Figure 3:**
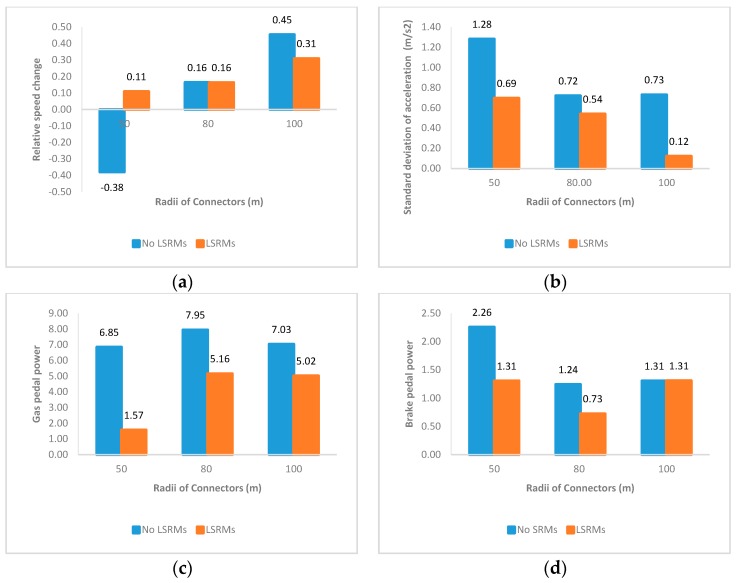
Mean values of four indicators of all subjects in the entire connector: (**a**) Relative speed change; (**b**) standard deviation of acceleration; (**c**) gas pedal power; and (**d**) brake pedal power.

In this part, profiles of four indicators in the entire interchange connector are shown in Figure 3, and the following findings are obtained:

1 From Figure 3a, it was obvious that the relative speed changes in connectors paved with LSRMs in all three scenarios were positive; that is, LSRMs failed to make participants slow down when they exited the connector. Meanwhile, in Figure 3d, the brake pedal forces related to LSRMs were also lower than No LSRMs, which meant that LSRMs also seemed to be ineffective in prompting participants to apply more force on the brake pedal.
2 In Figure 3b,c, it seemed that both standard deviations of acceleration and gas pedal powers in interchange connectors paved with LSRMs in all three scenarios were lower than No LSRMs. It was assumed that LSRMs would have better effects on the adaptability and driver’s gas pedal performance.
3 Comparing between Figure 3c,d, it was apparent that gas pedal power and brake pedal power in connectors without LSRMs were higher than the ones with LSRMs. According to the definitions mentioned above, lower power might refer to lower press intensity, shorter press duration time, less press frequencies, or the interaction effects. Based on this, it was implied that drivers would conduct more operating performances in connecters without LSRMs, while provide less power in connectors with LSRMs. It implied that LSRMs would control driver’s gas and brake pedal use to some extent.

### 3.2. Continuous Sections

**Figure 4:**
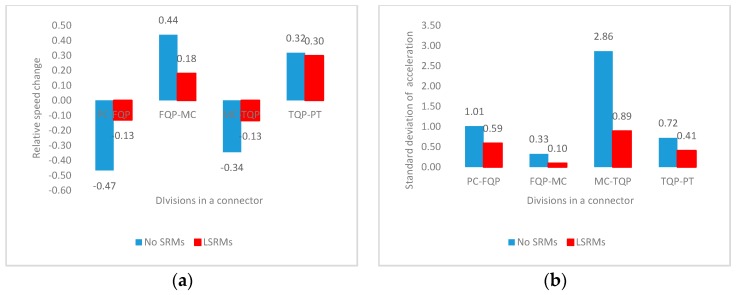

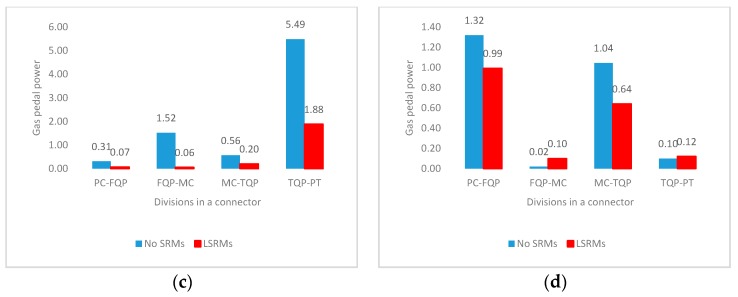
Mean values of four indicators of all subjects in continuous four divisions of connectors with 50-m radius: (**a**) Relative speed change; (**b**) standard deviation of acceleration; (**c**) gas pedal power; and (**d**) brake pedal power.

Analytical results in Figure 3 reflected a holistic view of vehicle operation and driver’s operating performance in an entire interchange connector. However, the effects of LSRMs on vehicle maneuvering and driver’s operating performance might vary at different locations in a connector. Figure 4 described the change patterns of four indicators in connectors with 50-m radius. It revealed that all of four indicators would change in continuous sections in connectors. For example, comparing between Figure 3a and Figure 4a, although the drivers finally increased speed in connectors with 50-m radius and LSRMs, they would still decelerate in the PC-FQP and MC-TQP sections. Thus, the conclusions about the effectiveness of LSRMs in interchange connectors could not be directly summarized based on the analytical results of entire connector section. In the following texts, all of four indicators in each section were independently analyzed, in order to better obtain the effects of LSRMs in different divisions in one connector, and provide more precise advices of the deployment of LSRMs.

#### 3.2.1. The PC-FQP Section

**Figure 5:**
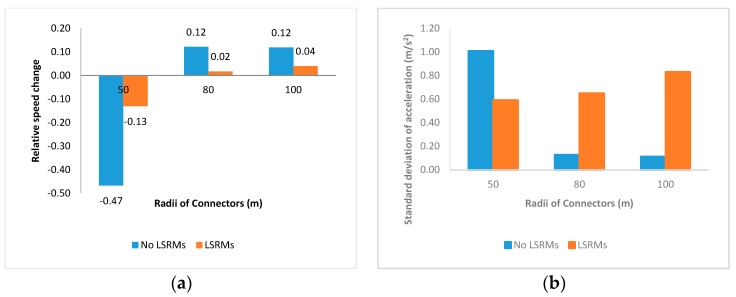

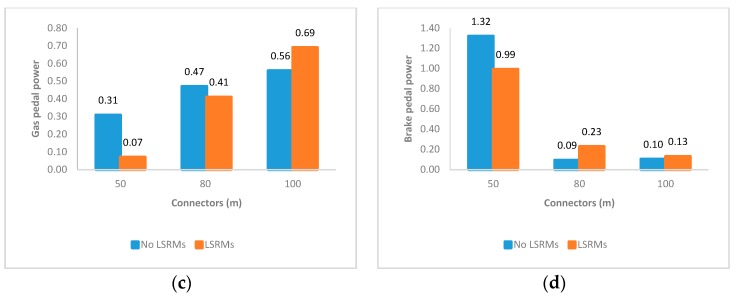
Mean values of four indicators of all subjects in the PC-FQP section: (**a**) Relative speed change; (**b**) standard deviation of acceleration; (**c**) gas pedal power; and (**d**) brake pedal power.

As mentioned above, interchange connectors in virtual scenarios were divided into four even sections, in order to thoroughly observe the effects of LSRMs on driving behavior in interchange connectors. The PC-FQP section was the first part of the connector, and Figure 5 illustrates the effects of LSRMs in this section:

1 From Figure 5a, it is obvious that participants decelerate in interchange connectors with a radius of 50 m and speed up in connectors with a radius of 80 m and 100 m. In addition, considering the speed reduction in connectors paved by LSRMs was smaller than the one in connectors with no LSRMs in the 50-R scenario, it implied that sharp curve might have better effects on making drivers decelerate than LSRMs.
2 In Figure 5b, without LSRMs, it seemed that participants would drive more smoothly in connectors with larger radii. On the contrary, participants drove more smoothly in connectors with LSRMs than in connectors without LSRMs when the radius was 50 m; nevertheless, this smoothness weakened as the increase of radii. Compared with the results of relative speed change in the same section, it was implied that LSRMs would have better effectiveness and adaptability in the PC-FQP section when the radius was 50 m.
3 In Figure 5c,d, it was apparent that subjects would press harder on the gas pedal and apply less pressure to the brake pedal when the radius of the interchange connectors increased, regardless of presence of LSRMs. Similar to (1) and (2), it could be inferred that LSRMs also have better effects on operation power in the first section of interchange connectors when the radius was 50 m, which further implies that LSRMs would influence subjects’ decelerating consciousness in the first section with a 50-m radius. Compared to Figure 5a, however, it seemed that such influence on decelerating consciousness did not bring speed reduction.

#### 3.2.2. The FQP-MC Section

**Figure 6:**
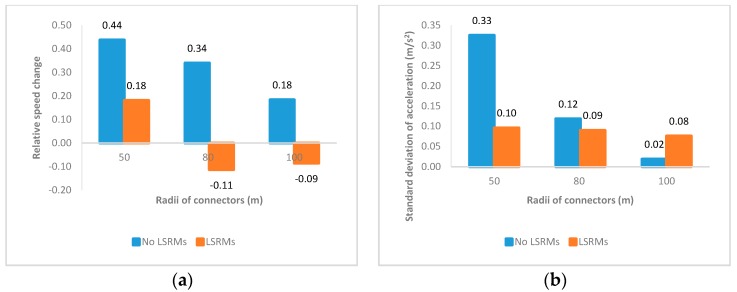

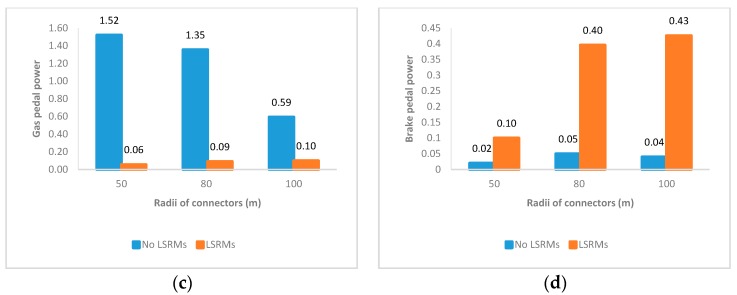
Mean values of four indicators of all subjects in the FQP-MC section: (**a**) Relative speed change; (**b**) standard deviation of acceleration; (**c**) gas pedal power; and (**d**) brake pedal power.

The FQP-MC section was the second part of the interchange connectors. Figure 6 describes the effects of LSRMs in this section, which seem to show the opposite effects as compared to the first section:

1 In Figure 6a, participants tended to speed less in the 80-R and 100-R scenarios without LSRMs, and even reduced speeds in the 80-R and 100-R scenarios with LSRMs. Combining with Figure 4a, this could imply that LSRMs would make subjects decelerate in the first quarter section when the radius was 50 m, and in the second quarter section when the radius was 80 m and 100 m. Besides, LSRMs would also restrain drivers from speeding more in the FQP-MC section.
2 In Figure 6b, the trend of standard deviation of acceleration in the FQP-MC section without LSRMs was similar to the trend shown in Figure 5b, while LSRMs might have no effects on drivers’ adaptability, regardless of the radius.
3 In Figure 6c,d, when the interchange connector was not paved with LSRMs, participants preferred to press gas pedal, rather than the brake pedal, and provided less power on the gas pedal with the increase in radii. On the contrary, LSRMs would make drivers focus more on the brake pedal to reduce speed, and drivers provided more power on the brake pedal when the radii were 80 m and 100 m, which was identical to (1).

#### 3.2.3. The MC-TQP Section

**Figure 7:**
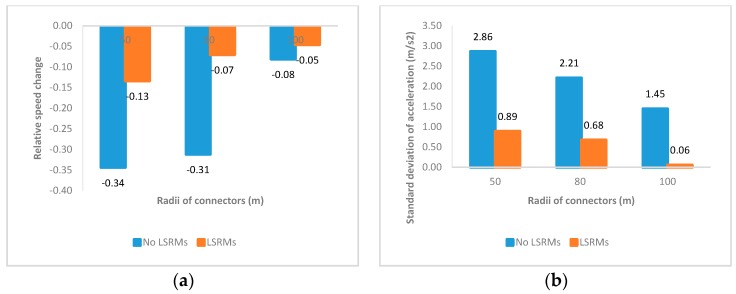

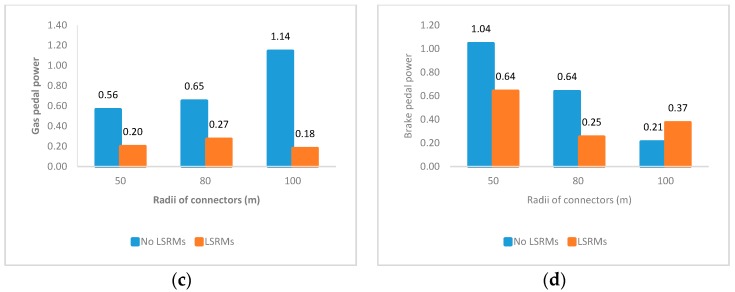
Mean values of four indicators of all subjects in the MC-TQP section: (**a**) Relative speed change; (**b**) standard deviation of acceleration; (**c**) gas pedal power; and (**d**) brake pedal power.

The MC-TQP section was the third quarter section in the interchange connectors. Figure 7 shows the effects of LSRMs in this section:

1 In Figure 7a, participants decelerated in the third quarter section of interchange connectors, regardless of the radius and whether LSRMs were installed. As the radius increased, the extent of decelerating decreased. Moreover, it seemed that the deceleration with LSRMs was weaker than the deceleration without LSRMs. However, it did not mean that LSRMs have lost effectiveness in this section. In fact, in Figure 7b, we could see that the standard deviation of accelerations in connectors with LSRMs were lower than the ones with No LSRMs. Therefore, it was probably that LSRMs would make drivers decelerate and maintain a steady state in the MC-TQP section.
2 In Figure 7c,d, subjects conducted much more operating performances in the MC-TQP section without LSRMs, and provided less force on the gas pedal and brake pedal in this section with LSRMs, which led to relatively low speed change and low standard deviation of acceleration. Such characteristics of pedal use contributed to the obvious speed change and high standard deviation of acceleration in this section without LSRMs. On the contrary, paved with LSRMs, subjects would travel in relatively low speed and perform less adjustment of gas and brake pedal, which would guarantee subject’s safety and comfort.

#### 3.2.4. The TQP-PT Section

**Figure 8:**
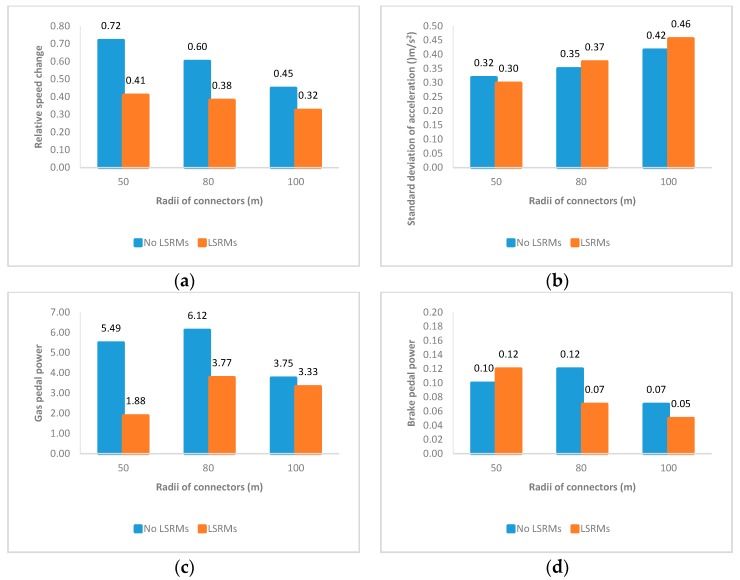
Mean values of four indicators of all subjects in the TQP-PT section: (**a**) Relative speed change; (**b**) standard deviation of acceleration; (**c**) gas pedal power; and (**d**) brake pedal power.

The TQP-PT section was the final part of the interchange connectors. Figure 8 describes the effects of LSRMs in this section. In Figure 8a–d, although LSRMs did affect vehicle maneuvering and drivers’ operation performance, we could see that participants would choose to accelerate and press harder on the gas pedal, while applying less force to the brake pedal, regardless of the radius and presence of LSRMs, in the final quarter of the connectors.

### 3.3. Statistical Analysis in Four Sections

According to the experimental design in this research, analysis of variance with repeated measures (rANOVA) and contrast analysis (S-N-K method) were used to evaluate the effects of LSRMs on driving behavior in different sections on interchange connectors with different radii. Test results revealed that there were significant main effects of fragment sections on relative speed change (F_(3,174)_ = 6.102; *p* < 0.05), standard deviation of acceleration (F_(3,174)_ = 4.298; *p* < 0.05), gas pedal force (F_(3,174)_ = 17.104; *p* < 0.05), and brake pedal force (F_(3,174)_ = 3.478; *p* < 0.05). Statistically analytical results in detail are summarized in Table 2. The symbol “√” means that the effect of LSRMs is statistically significant and better than No LSRMs, while the symbol “×” means that the effect of No LSRMs is statistically significant and worse than LSRMs; the blank means that there are no significant differences between the effects of No LSRMs and LSRMs. From Table 2, we could conclude that:

1 LSRMs could significantly reduce vehicles’ travel speed in the second and the final section of interchange connectors. Although LSRMs could make drivers decelerate in the first and third quarter sections of the connectors (see Figure 4a and Figure 6a), the effects of LSRMs were significantly smaller than No LSRMs. However, we could still conclude that LSRMs were able to control speed to different degrees in interchange connectors.
2 In the PC-FQP, FQP-MC, and MC-TQP sections, LSRMs could significantly enhance drivers’ adaptability when the radius was 50 m.
3 In all four sections, LSRMs could also significantly control drivers’ gas pedal operation; particularly, this phenomenon existed in the FQP-MC and MC-TQP sections with all three radii.
4 Compared to gas pedal power, LSRMs could only prompt drivers to apply more brake pedal operations in the FQP-MC section, when the radii were 80 m and 100 m.

**Figure 9:**
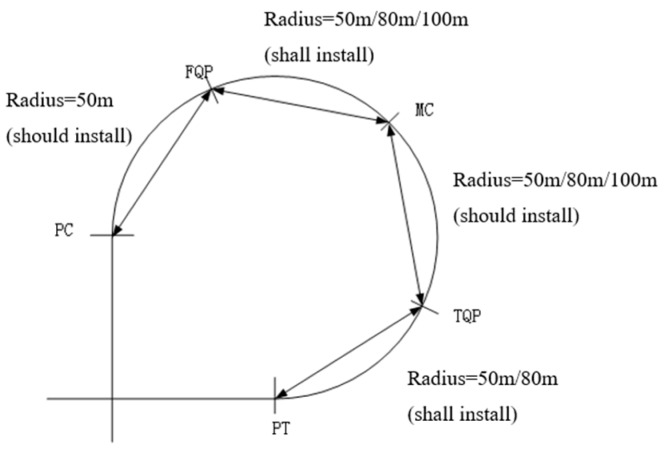
Guidance for implementing LSRMs on interchange connectors.

According to the analytical results shown in Figure 5, Figure 6, Figure 7 and Figure 8 and Table 2, Figure 9 illustrates a series of theoretical recommendations of LSRMs installation in continuous sections with different radii:

1 In the PC-FQP section, from the aspect of vehicle maneuvering and driver’s brake pedal use, there might be no need to pave LSRMs in this section, regardless of the radius; however, referring to driver’s adaptability and gas pedal use, SRMs could be deployed in this section when the radius was 50 m. In other words, LSRMs are needed in the PC-FQP section of 50 m-radius connectors, and are not needed for sections with a radius of 80 m and above.
2 In the FQP-MC section, LSRMs could significantly make drivers decelerate, improve driver’s adaptability or limit driver’s gas pedal and brake pedal use, although such effects were different for different radii. Therefore, LSRMs are recommended for the FQP-MC sections with a radius of 50 m, 80 m or 100 m.
3 In the MC-TQP section, similar to (1), if the intention is to force drivers to slow down, LSRMs are not recommended to be used in this section; if the primary goal is to improve driver’s adaptability and limit driver’s gas pedal use, LSRMs could be considered to be deployed in this section regardless of radii. Thus, LSRMs should be used in the MC-TQP section when the radius is 50 m, 80 m or 100 m.
4 In the TQP-PT section, LSRMs should be used when the radius is 50 m or 80 m, since LSRMs could significantly control speed and driver’s gas pedal use.

## 4. Discussion and Future Work

This paper evaluated the effect of LSRMs on driving behavior, including vehicle maneuvering and drivers’ operation performance on interchange connectors with different radii. The experiment was performed in a driving simulator.

Since the order of the two LSRMs conditions (No LSRMs and LSRMs) in the three scenarios was the same, this could lead to a learning effect. Therefore, as illustrated in Section 2.3, especially in Figure 2a, one-kilometer tangents, which connected two connectors related to different LSRMs conditions, were designed to fade subjects’ memory or impression of the previous connector section, so as to eliminate or reduce the learning effect. In fact, the average and standard deviation of speeds at the beginning of the interchange connectors of all subjects are listed in Table 3. It is apparent that the average and standard deviation of speeds at the beginning of the interchange connectors with No LSRMs and LSRMs are approximately the same in the same scenario, implying that the learning effect is controlled, so that it does not substantially influence the results and conclusions. In addition, the analysis of variance with repeated measures (rANOVA) is commonly used to validate the significant effects of treatments in repeated measures. Since the repeated measures were usually organized in the same time-sequential order for all subjects, rANOVA was conducted to evaluate the effectiveness of LSRMs.

This research mainly focused on the effectiveness of LSRMs in interchange connectors. In fact, nearly all ramps on interchanges have been paved with LSRMs, though those installations were usually made on the basis of engineers’ judgment and experiences, since insufficient theoretical support exists to provide guidance. Besides, the interchange connectors discussed in this paper were all downhill, and there are still many uphill connectors paved with LSRMs. Therefore, in future studies, it is necessary to study the effects of LSRMs on other types of ramps on interchanges, and consider the differences between uphill and downhill connectors; furthermore, this research will be extended to other ramps or curves on urban roads and highways.

The effectiveness and adaptability of both kinds of SRMs in downhill sections in urban expressways have been evaluated [7,8,9]. In those studies, the effects of LSRMs on speed reduction and drivers’ operation performance were significantly worse than TSRMs. Nevertheless, as shown in Table 2, LSRMs had various impacts on vehicles and drivers in interchange connectors. It is likely that the specific road conditions in interchange connectors would strengthen the visual illusions generated by LSRMs. Thus, researchers will also study the mechanisms of LSRMs, which can further guide the design principles of LSRMs and increase their effectiveness.

## 5. Conclusions

This paper evaluated the effects of LSRMs on driving behavior in interchange connectors with various radii. By analyzing the data collected in a driving simulator, this research has reached the following conclusions:

LSRMs could reduce vehicles’ travel speed and limit drivers’ willingness to speed up in the entire interchange connector when the radius was 50 m, and such effects were stronger in the second and the final section of the connectors. When the radii were 80 m and 100 m, LSRMs tended to affect speed or restrain drivers’ willingness to accelerate in the last three quarters of the connectors.
LSRMs could enhance drivers’ adaptability in the first three quarters of the interchange connectors when the radius was 50 m, and could also make driver’s travel more comfortably in the third quarter section of the connector with all three radii.
LSRMs could control driver’s gas pedal operation throughout the entire connector when the radius was 50 m. On the other hand, LSRMs also prompted drivers to apply less force on gas pedal in the last three quarters of the interchange connectors when the radii were 80 m and 100 m.
LSRMs only prompted drivers to conduct more brake pedal operations in the second section, when the radii were 80 m and 100 m.
The second quarter section of the connector—the FQP-MC section—seemed to be the key section in an interchange connector, on the basis of this experimental condition, where LSRMs have better effects on influencing vehicle maneuvering and drivers’ operation performance.
On the one hand, LSRMs should be used in the first quarter section of connectors when the radius was 50 m, and in the third quarter section regardless of radii. On the other hand, LSRMs should be used in the second quarter section regardless of radii, and in the final quarter section when the radii were 50 m and 80 m.

In summary, this paper discussed the effects of LSRMs placements in urban interchange connectors with various radii, based on a driving simulator experiment. In future research, in order to develop guidelines about optimal placement of LSRMs, the relationship between LSRMs, road alignments of the interchange connectors or curves, and vehicle maneuvering and drivers’ operation performance needs to be further evaluated.

## Figures and Tables

**Table 1:** Alignment parameters of virtual scenarios.

Scenario No.	Radii (m)	Length of Connector (m)	Length of Entire Scenario (m)	Length of Each Section (m)	Lane Width (m)
1	50	235.5	2971.0	58.88	3.75
2	80	376.8	3253.6	94.20	
3	100	471.0	3442.0	117.75	

**Table 2:** Statistical results of four indicators in continuous sections with different radii (*p* < 0.05).

Indicators	PC-FQP			FQP-MC			MC-TQP			TQP-PT		
	50 m	80 m	100 m	50 m	80 m	100 m	50 m	80 m	100 m	50 m	80 m	100 m
Relative speed change	×			√	√	√		×	×	√	√	
Standard deviation of acceleration	√	×	×	√			√	√	√			
Gas pedal force	√			√	√	√	√	√	√	√	√	
Brake pedal force	×				√	√	×	×				

Notes: PC-FQP: From the point of connector to the first quartile point; FQP-MC: From the first quartile point to the middle point of connector; MC-TQP: From the middle point of connector to the third quartile point; TQP-PT: From the third quartile point to the point of tangent; ×: The effect of No LSRMs is statistically significant and worse than LSRMs; √: The effect of LSRMs is statistically significant and better than No LSRMs.

**Table 3:** The average and standard deviation of speeds at the beginning of connectors with different longitudinal speed reduction markings (LSRMs) conditions in all scenarios (in km/h).

Radii	No SRMs		LSRMs	
	Mean	Std.	Mean	Std.
50 m	54.9	17.7	54.7	19.1
80 m	56.5	15.1	57.9	18.2
100 m	60.2	18.2	59.1	17.3

Notes: LSRMs: Longitudinal speed reduction markings; SRMs: Speed reduction markings; Std.: Standard deviation.
